# Cytokine clearance in serum and peritoneal fluid of patients undergoing damage control surgery with abdominal negative pressure therapy for abdominal sepsis

**DOI:** 10.1515/pp-2020-0122

**Published:** 2020-12-29

**Authors:** Seraina Faes, Martin Hübner, Nicolas Demartines, Dieter Hahnloser

**Affiliations:** Department of Visceral Surgery, Lausanne University Hospital CHUV, University of Lausanne (UNIL), Lausanne, Switzerland

**Keywords:** abdominal negative pressure therapy, acute abdomen, cytokine clearance, damage control surgery, emergency surgery

## Abstract

**Objectives:**

Open abdomen technique with negative pressure therapy (NPT) is widely used in patients with severe abdominal sepsis. The aim of this study was to evaluate cytokine clearance in serum and peritoneal fluid during NPT.

**Methods:**

This prospective pilot study included six patients with severe abdominal sepsis requiring discontinuity resection and NPT for 48 h followed by planned reoperation. Cytokines (IL6, IL8, IL10, TNFalpha, and IL1beta) were measured in the serum and peritoneal fluid during index operation, on postoperative days 0, 1, and 2.

**Results:**

Concentrations of cytokines in peritoneal fluid were higher than in serum. IL10 showed a clearance both in serum (to 16.6%, p=0.019) and peritoneal fluid (to 40.9%, p=0.014). IL6 cleared only in serum (to 24.7%, p=0.001) with persistently high levels in peritoneal fluid. IL8 remained high in both serum and peritoneal fluid. TNFalpha and IL1beta were both low in serum with wide range of high peritoneal concentrations. Only TNFalpha in peritoneal fluid showed significant differences between patients with ischemia *vs.* perforation (p=0.006).

**Conclusions:**

The present pilot study suggests that cytokines display distinct patterns of clearance or persistence in the peritoneal fluid and serum over the first 48 h of treatment in severe abdominal sepsis with NPT.

## Introduction

Abdominal sepsis is associated with significant morbidity and mortality due to multiorgan failure caused by systemic inflammatory response [1], [2], [3]. In abdominal sepsis, a local source such as hollow organ perforation or mesenteric ischemia results in local and systemic inflammatory response. Pathophysiologically, the loss of intestinal continuity in the case of intestinal perforation or the loss of barrier function of the bowel wall in the case of mesenteric ischemia lead to an active secretion of pro-inflammatory-mediator-rich ascites by the peritoneum [4], [5], [6], [7], [8]. Studies have indicated that local inflammatory response may be stronger than systemic inflammatory response in the case of abdominal sepsis [9], [10], [11], [12]. Hence, the inflammatory ascites is suspected to be an important motor for systemic inflammation and hence an important determinator of multiorgan failure [4], [9], [10], [13], [14].

Cytokines are regulators of acute or chronic infection and immune response. The pro-inflammatory cytokine Interleukin 6 (IL6) is a potent mediator of sepsis and is associated with an increased risk of organ dysfunction and mortality in patients with abdominal sepsis [15], [16], [17], [18], [19], [20]. Furthermore, IL6 was shown to reflect overall cytokine cascade activation [21]. Interleukin 8 (IL8) is a chemokine produced by macrophages, but also epithelial and endothelial cells, mediates chemotaxis of neutrophils and stimulates phagocytosis in the innate immune response [22]. Oxidative stress is a potent inducer of IL8 secretion, making it a key pro-inflammatory mediator of localized inflammation [23]. IL10 is an anti-inflammatory cytokine that enhances antibody response by CD4+ T-cell activation, costimulatory molecules on macrophages and suppression of secretion of pro-inflammatory cytokines triggered by bacterial products and lipopolysaccharide [24], [25], [26], [27], [28]. It is elevated post abdominal surgery and decreases in the postoperative course of patients without complications [29]. Tumor necrosis factor alpha (TNFalpha) is a pro-inflammatory cytokine and enhances cellular immune response. TNFalpha levels are elevated in intra-abdominal sepsis [30]. Levels decrease in patients without postoperative complications, whereas patients with complications show a secondary rise preceding the complication [29]. Interleukin 1 beta (IL1beta) is an important pro-inflammatory cytokines modulating monocyte activation and pro-inflammatory signaling. It was shown to be elevated in patients with gram-negative bacteremia and correlate with the development and severity of sepsis, multiorgan failure, death, and sepsis susceptibility [31], [32], [33].

Open abdomen technique is widely used in patients with severe abdominal sepsis to avoid abdominal compartment syndrome and facilitate 2nd look laparotomy [34], [35], [36], [37], [38], [39]. After damage control surgery (discontinuity resection of affected bowel), the abdomen is temporarily closed to provide visceral coverage [38], [40], and the patient stabilized on the ICU. The patient is taken back to the operating room 48 h later to restore intestinal continuity. Several groups including our institution adopted this systematic 2nd look strategy in unstable patients with abdominal sepsis [36], [41], [42], [43], [44], [45], [46], [47]. In addition to simple visceral coverage, a temporary abdominal closure using negative pressure therapy (NPT) might help clearing pro-inflammatory cytokines and potentially reducing peritoneal and systemic inflammation and thus improving outcomes [9], [13], [14], [48], [49], [50], [51], [52]. However, little is known about clearance of cytokines from serum and peritoneal fluid under this therapy.

The aim of this pilot study was to describe and compare cytokine concentrations in serum and peritoneal fluid in the early postoperative period in patients with abdominal sepsis and NPT. The study explores the following questions (I) are cytokine concentrations in the peritoneal fluid higher than in the serum, (II) do cytokines have similar kinetics in the peritoneal fluid and serum, and (III) are kinetics of clearance of cytokines dependent of the source of abdominal sepsis.

## Materials and methods

This prospective pilot study included six patients with severe abdominal sepsis requiring discontinuity resection and abdominal NPT for 48 h followed by 2nd look laparotomy with planned bowel reconstruction. The study was funded by an educational grant from the University Hospital; no industry sponsored funds were received. Funding was available for six patients.

The five cytokines IL6, IL8, IL10, TNFalpha, and IL1beta were chosen due to their important role in the inflammatory response to intra-abdominal sepsis. Peritoneal levels were obtained in order to determine local inflammatory response and serum levels in order to determine systemic inflammatory response. Cytokines were measured in the serum and peritoneal fluid during index operation, upon arrival in intensive care unit (ICU) on postoperative day (POD) 0, on POD1, and on POD2. Peritoneal fluid was acquired by sampling intra-abdominal fluid at the initiation of index operation (timepoint 1) and by the aspiration of peritoneal liquid from the abdominal vacuum container (timepoints 2 to 4). Blood samples were taken from venous blood draws at the same timepoints. Cytokines were analyzed in triplicates by Enzyme Linked ImmunoSorbent Assay (ELISA).

In addition to cytokines, laboratory parameters including electrolytes, glucose and proteins were measured in serum and peritoneal fluid, as well as CRP, procalcitonin, and lactate in the serum at the four timepoints. Baseline characteristics of patients including age, sex, American Society of Anesthesiologists (ASA) score, Acute Physiology And Chronic Health Evaluation (APACHE)-II score, and Sequential Organ Failure Assessment (SOFA) score, as well as clinical outcome including length of hospital stay, length of ICU stay, 30-day mortality, and comprehensive complication index were registered.

Individual values of cytokines were plotted in spaghetti plots and compared for peritoneal and serum values. Variability between patients (intersubject variability) was calculated for each cytokine. Cytokine clearance was described as difference in percentage (%) from baseline (timepoint 1, index operation) to timepoints 2–4 (POD0, POD1, and POD2) and plotted in bar charts. Differences in clearance patterns over all timepoints were calculated. In a subanalysis, patients with mesenteric ischemia and bowel perforation were compared with respect to reduction of cytokine levels from timepoint 1 to timepoint 4 (ΔPOD2) for both peritoneum and serum. Similarly to cytokines, additionally measured laboratory parameters were plotted in spaghetti plots and compared for their peritoneal and serum values. In accordance to cytokines, difference between ischemia and perforation was calculated for these additional laboratory parameters.

### Statistics and analysis

Continuous parametric variables were reported as mean ± SD and compared by using unpaired student’s t-test (two-tailed, *α* of 0.05, confidence level 95%) in the case of absence of additional timepoint factor or by using two-way ANOVA test (*α* of 0.05, confidence level 95%, Geisser-Greenhouse correction) for additional timepoint comparison with ischemia and perforation as column factors (3 subjects as subcolumns) and timepoints as row factors, and by using ANOVA multiple comparisons (Turkey correction) to compare two individual timepoints. Continuous nonparametric variables were reported as median + range and compared by using Mann-Whitney test (unpaired, two-tailed, α of 0.05, confidence level 95%). Categorical variables were reported as frequency (%) and compared by use of Fisher’s exact test (two-sided, *α* of 0.05, confidence level 95%). Statistical analysis and graphs were performed by using GraphPad Prism 8.3.0.

## Results

The study cohort included three patients with intestinal perforation and three patients with mesenteric ischemia. Pertinent baseline demographics and outcomes did not significantly differ for these two groups and are detailed in Table 1. Two patients in the perforation group died, one patient in septic shock on POD2 before 2nd look laparotomy and intestinal reconstruction, and one on POD15 due to respiratory decompensation. According to the institutional treatment algorithm, four patients had intestinal reconstruction on POD2 and one received a terminal ileostomy after initial total colectomy, as an ileorectal anastomosis was not considered beneficial for this patient.

**Table 1:** Baseline characteristics and clinical outcomes of patients with negative pressure therapy for abdominal sepsis.

Diagnosis	Ischemia	Ischemia	Ischemia	Perforation	Perforation	Perforation
**Age, years**	80	46	78	84	85	87
**Sex**	Male	Male	Male	Female	Male	Female
**ASA score**	4	4	3	4	4	4
**APACHE-II score**	35	30	31	27	26	29
**SOFA score**	14	10	11	11	10	11
**Length of stay, days**	32	67	13	2	64	15
**Length of ICU stay, days**	15	20	3	2	6	4
**30-day mortality**	0	0	0	1	0	1
**Comprehensive complication index**	46.5	47.2	8.7	100	22.6	100

Each column represents one patient. ASA, American Society of Anesthesiologists; APACHE, Acute Physiology And Chronic Health Evaluation; SOFA, Sequential Organ Failure Assessment; ICU, intensive care unit.

**Figure 1:**
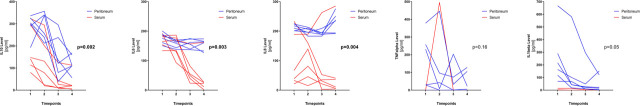
Cytokine levels in peritoneal fluid and serum of patients under negative pressure therapy. Spaghetti plots for levels of cytokines IL10, IL6, IL8, TNFalpha, and IL1beta are depicted for each timepoint (1=index operation, 2=POD0 arrival ICU, 3=POD1, 4=POD2). Each line represents one patient with blue lines corresponding to peritoneal fluid levels and red lines corresponding to serum levels. p-Values compare peritoneal and serum levels for all six patients with respect to their timepoints.

Early postoperative course of cytokines revealed higher values in peritoneal fluid compared to serum concentrations (Figure 1). Values of serum IL8 (p<0.0001) and peritoneal IL1beta (p=0.004) were marked by a high intersubject variability.

**Figure 2:**
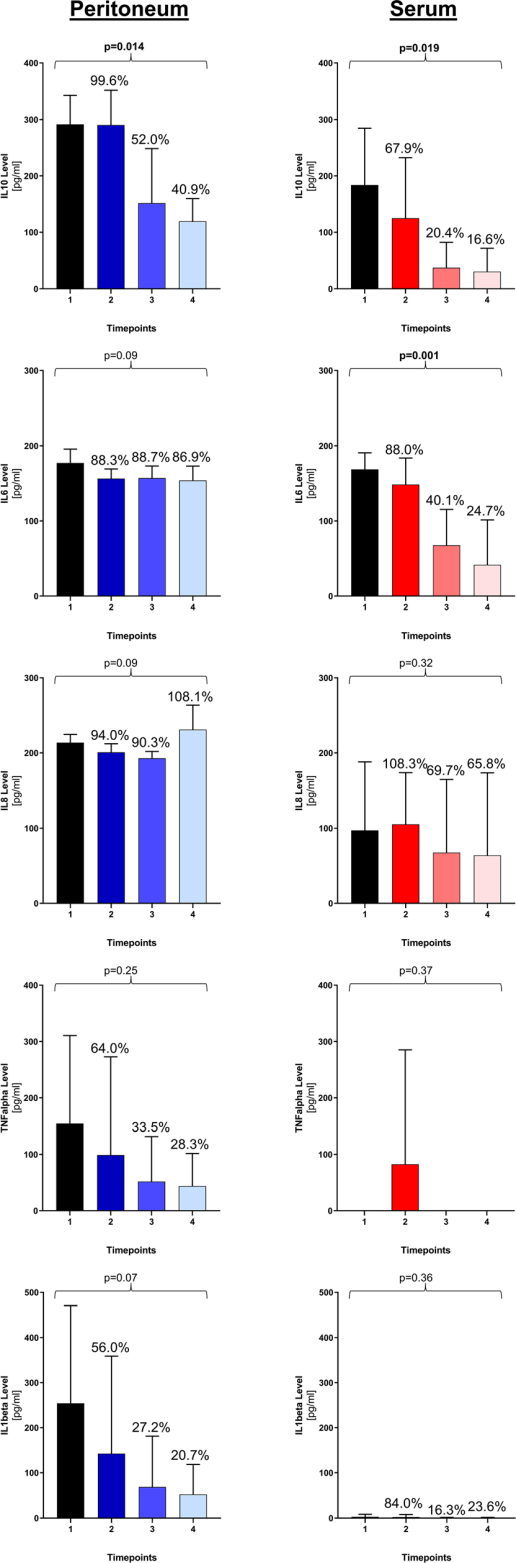
Clearance pattern of cytokines in peritoneal fluid and serum. For each cytokine, means and standard deviations are represented for each timepoint with their corresponding percentages in relation to timepoint 1 (index operation). p-Values are calculated comparing kinetics over all four timepoints.

A kinetics of clearance was found for IL10 in the serum (p=0.019) and peritoneal fluid (p=0.014) and IL6 in the serum (p=0.001), while peritoneal IL6 and peritoneal IL8 levels remained persistently high (Figure 2). Serum IL8 levels decreased in five patients but increased in one patient. This was the patient who deceased on POD2. Serum values of TNFalpha and IL1beta were very low or even undetectable at most timepoints, which was not the case for their peritoneal counterparts.

**Figure 3:**
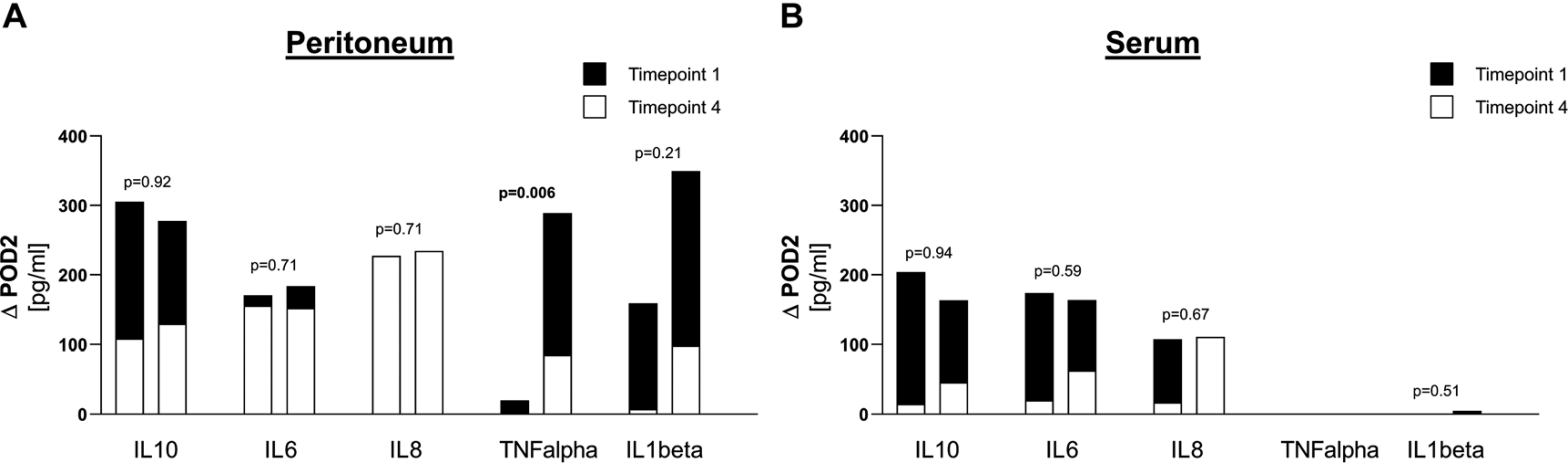
Comparison of reduction in cytokine levels between ischemia and perforation. (A) Values for peritoneum, (B) Values for serum. For each cytokine, means are represented for timepoint 1 (index operation) in black and for timepoint 4 (POD2) in white, in order to display the reduction between timepoint 1 and timepoint 4 (ΔPOD2). For each cytokine, left bar corresponds to ischemia and right bar corresponds to perforation. p-Values compare ΔPOD2 between ischemia and perforation.

When comparing patients with ischemia to patients with perforation with respect to reduction of cytokine level between timepoint 1 and timepoint 4 (ΔPOD2), only peritoneal TNFalpha levels showed a significant difference of ΔPOD2 for the two groups (Figure 3).

In contrast to cytokines with higher concentrations in the peritoneal fluid as compared to the serum, electrolytes (sodium p=0.07 and potassium p=0.96) in the peritoneal fluid and serum were not significantly different, and values for glucose (p=0.008), protein (p<0.0001), and albumin (p<0.0001) were even higher in the serum. On the other hand, chloride (p=0.019) and lactate dehydrogenase (LDH) levels (p=0.0007) were higher in the peritoneal fluid than in the serum. None of the aforementioned additionally measured laboratory parameters was significantly different between ischemia and perforation.

Maximal values for CRP were noted on POD1 (mean 289 mg/L (SD 56 mg/L) for ischemia and mean 247 mg/L (SD 111 mg/L) for perforation) and were not significantly different for the two groups (p=0.97 for peak values and p=0.95 for all timepoints). CRP was the only marker to show an increasing kinetics over the four timepoints (p=0.002). The other additionally measured parameters including glucose, sodium, potassium, chloride, protein, albumin, LDH, and amylase had no statistically significant kinetics over the first 48 h. Procalcitonin peaks found on POD1 (mean 9.4 μg/L (SD 10.1 μg/L) for ischemia and mean 25.5 μg/L (SD 18.8 μg/L) for perforation) also did not differ significantly between the two groups (p=0.74 for peak values and p=0.11 for all timepoints). Lactate peaks were found during index operation and were not significantly different in the ischemia and perforation group (3.1 mmol/L (SD 1.3 mmol/L) vs. 3.7 mmol/L (SD 0.8 mmol/L); p=0.95 for peak values and p=0.35 for all timepoints).

## Discussion

Pro-inflammatory cytokines induced by different endo- or exogenous triggers play an important role in initiating inflammatory response and regulating host defense. In abdominal sepsis, this cytokine cascade is initiated upon a direct toxic effect of pathogens like bacteria and intestinal fluid after bowel perforation or upon release of toxic metabolites like lactate, reactive oxygen metabolites, and nitric monoxide in mesenteric ischemia. The pro-inflammatory cytokine release serves to contain and resolve the inflammatory focus through the activation of local and systemic inflammatory responses, however, an overexpression of pro-inflammatory cytokines contributes to the development of organ dysfunction and poor outcomes. This pilot study provides information on local and systemic levels and clearance patterns of cytokines in patients with abdominal sepsis requiring bowel discontinuity resection and undergoing negative pressure therapy.

Cytokine-mediated inflammatory response was compartmentalized, with a higher local response in the peritoneal cavity compared to the systemic response in the present study. Similarly, higher peritoneal cytokine concentrations were measured in patients undergoing planned relaparotomy for severe secondary peritonitis with a 19-fold higher concentration of TNFalpha and a 993-fold higher concentration of IL6 in the peritoneal exudate compared to the serum [9]. A study analyzing systemic and peritoneal cytokines levels (in the liquid of peritoneal drain) after abdominal surgery for diverse indications showed significantly higher TNFalpha, IL6, and IL10 peritoneal values [10]. Another study, also sampling peritoneal fluid from abdominal drains of patients undergoing elective intestinal resections or pancreatic surgery [11] displayed high peritoneal TNF alpha and peritoneal IL1beta concentrations within the first 12 h after operation, while serum concentrations were undetectable. The same was observed in the present study. Apart from one patient, all serum TNF alpha and INF1 beta levels were undetectable or near undetectable. Furthermore, an enhanced peritoneal inflammatory response following gut ischemia reperfusion was shown in animal reperfusion models [12]. These data suggest that the compartmentalization for TNFalpha and IL1beta is even stronger than for the other measured interleukins, and that the peritoneum acts as barrier for cytokine control. Studies have suggested peritoneal mesothelial cells as well as peritoneal macrophages and leukocytes as important sources of intra-abdominal cytokine release [7], [8]. A specific immune cell recruitment postsurgery with respect to natural killer cells, monocytes, natural killer T cells, and CD5+ B cells in the peritoneal fluid and blood has been demonstrated [53].

The present pilot study suggested distinct clearance patterns for different cytokines with a rapid IL10 clearance in both peritoneum and serum, an IL6 clearance in the serum only, as opposed to persistently high levels in the peritoneum and a serum clearance of IL8 in five out of six patients, with the patient deceased shortly after the last 48 h sampling timepoint having an increase of IL8 serum levels, whilst IL8 levels stayed high for all patients. A study with 19 patients undergoing open abdominal treatment for secondary peritonitis showed no correlation of serum and peritoneal cytokine concentrations over time [13]. Similar to the present data, in this study, peritoneal IL6 levels did not decrease within the first hours after lavage, and serum and peritoneal IL8 concentrations were stable at 24 h. However, whereas patients in the mentioned study showed no changes in IL10 levels over time, a continuous decrease for both peritoneal and serum levels was found in the present study. Although there were no statistically significant differences in cytokine concentrations for survivors vs. nonsurvivors, survivors had relatively large individual changes of peritoneal IL8 compared to non-survivors, which might go in line with the one patient in the present study showing serum IL8 increase associated with lethal outcome. An animal study on rats being injected with feces and bacteria intraperitoneally showed a strong correlation between peritoneal IL6 and IL10 levels with mortality [51], and a study on 17 patients undergoing planned relaparotomy for abdominal infection showed decreasing endotoxin and TNFalpha levels in survivors with persistent elevation in nonsurvivors [9].

Subgroup analysis in the present study suggested no difference of levels and clearance in patients with ischemia and perforation for all cytokines except TNFalpha levels which were significantly higher in patients with bowel perforation. Similarly, a study looking at intraperitoneal cytokine response after different indications for major abdominal surgery found higher peritoneal TNFalpha levels in patients undergoing rectal surgery [10]. An exogenous bacterial trigger as in the case of bowel perforation or rectal surgery possibly induces a stronger peritoneal TNFalpha release as opposed to endogenous metabolites released after mesenteric ischemia. Of note, TNFalpha is one of the earliest released pro-inflammatory mediator and possibly the only cytokine affected by this discrepancy as opposed to mediators released later in the cytokine cascade.

This study applied a negative pressure vacuum system for abdominal coverage after emergency abdominal discontinuity resection. A standardized damage control strategy in hemodynamically unstable patients and patients with acidosis or lactatemia due to abdominal sepsis is adopted in our institution. These patients undergo rapid damage control surgery with discontinuity resection followed by NPT and planned reoperation at 48 h. The role of NPT in cytokine clearance is not yet pertinently elucidated. Animal studies suggested an improved fluid clearance, reduced systemic inflammatory response, and better outcomes with NPT [14]. In humans, a study comparing ABThera vs. Baker’s vacuum pack with respect to blood and peritoneal cytokine levels after operation for abdominal injury or intra-abdominal sepsis did not show differences in plasma concentrations of different cytokines including IL6, IL1beta, IL8, IL10, and IL12 for the two comparative groups [48]. Patients of the ABThera group had, however, significantly lower 90-day mortality rates. A further study on patients with NPT with ABThera vs. Baker’s vacuum-packing displayed significantly higher 30-day primary fascial closure rates and lower 30-day mortality among patients for the ABThera group with a higher likelihood of survival in a multivariate logistic regression analysis [49]. A comparison of NPT vs. Bogota bag showed faster abdominal closure rate and earlier discharge from ICU for NPT [50]. Looking at vacuum-assisted closure vs. on demand laparotomy for secondary peritonitis, no difference in serum levels and clearance pattern of IL8 and peritoneal levels and clearance pattern of TNFalpha, as well as mortality was observed in the two groups [54]. It remains currently unknown if cytokines are directly related to improved outcomes.

In addition to cytokines, this study measured laboratory parameters, both in the peritoneum and serum. Sodium and potassium did not differ between serum and peritoneal fluids and protein and albumin levels were higher in the serum than the peritoneal liquid. The peritoneal liquid probably represents a transudate rather than exudate, and the liquid seems to be correctly evacuated by the vacuum system rather than concentrated in the abdominal cavity before evacuation. This would underline that the higher concentration of cytokines in the peritoneal fluid is not due to a concentration change of the liquid but an actual compartmentalization of cytokine release in the peritoneum.

Main limitation of this study is the small sample of only six patients. In fact, high variations between patients could be noted for some measurements. The present study has therefore to be regarded as pilot to provide baseline data.

In summary, high peritoneal cytokine concentrations and a distinct clearance of different cytokines were found both in serum and peritoneal fluid of patients with abdominal sepsis and intestinal discontinuity resection treated by negative pressure therapy. Further investigations are needed to evaluate (I) whether faster cytokine clearance associates with better clinical outcomes, (II) if NPT can help to improve cytokine clearance in serum and peritoneal fluid, and (III) if different triggers of abdominal sepsis have diverse effects on early cytokine cascade as opposed to later released metabolites.
